# Cannabinoid Receptor Agonist WIN55, 212-2 Attenuates Injury in the Hippocampus of Rats after Deep Hypothermic Circulatory Arrest

**DOI:** 10.3390/brainsci13030525

**Published:** 2023-03-22

**Authors:** Ming-Huan Yu, Qin Yang, You-Peng Zhang, Jia-Hui Wang, Ren-Jian-Zhi Zhang, Zhi-Gang Liu, Xiao-Cheng Liu

**Affiliations:** Department of Cardiovascular Surgery, TEDA International Cardiovascular Hospital, Chinese Academy of Medical Sciences, Graduate School of Peking Union Medical College, 61 Third Avenue, TEDA, Tianjin 300456, China

**Keywords:** deep hypothermic circulatory arrest, cannabinoid receptor, neuroprotection

## Abstract

Objectives: Postoperative neurological deficits remain a challenge in cardiac surgery employing deep hypothermic circulatory arrest (DHCA). This study aimed to investigate the effect of WIN55, 212-2, a cannabinoid agonist, on brain injury in a rat model of DHCA. Methods: Twenty-four male Sprague Dawley rats were randomly divided into three groups: a control group (which underwent cardiopulmonary bypass (CPB) only), a DHCA group (CPB with DHCA), and a WIN group (WIN55, 212-2 pretreatment before CPB with DHCA). Histopathological changes in the brain were evaluated by hematoxylin–eosin staining. Plasma levels of superoxide dismutase (SOD) and proinflammatory cytokines including interleukin (IL)-1β, IL-6, and tumor necrosis factor-alpha (TNF-a) were determined using an enzyme-linked immunosorbent assay (ELISA). The expression of SOD in the hippocampus was detected by Western blot and immunofluorescence staining. Levels of apoptotic-related protein caspase-3 and type 1 cannabinoid receptor (CB1R) in the hippocampus were evaluated by Western blot. Results: WIN55, 212-2 administration attenuated histopathological injury of the hippocampus in rats undergoing DHCA, associated with lowered levels of IL-1β, IL-6, and TNF-α (*p* < 0.05, *p* < 0.001, and *p* < 0.01, vs. DHCA, respectively) and an increased level of SOD (*p* < 0.05 vs. DHCA). WIN55, 212-2 treatment also increased the content of SOD in the hippocampus. The protein expression of caspase-3 was downregulated and the expression of CB1R was upregulated in the hippocampus by WIN55, 212-2. Conclusions: the administration of WIN55, 212-2 alleviates hippocampal injury induced by DHCA in rats by regulating intrinsic inflammatory and oxidative stress responses through a CB1R-dependent mechanism.

## 1. Introduction

Cerebral ischemic injury is one of the major causes of death and disability worldwide and represents a great burden to society [1], which may occur following cardiac arrest and cardiovascular surgery requiring cardiopulmonary bypass (CPB) [2,3]. Since being introduced in the 1970s, deep hypothermic circulatory arrest (DHCA) has become one of the most indispensable technologies in complex cardiac surgery to reduce the metabolic rate of tissues and organs to protect against ischemic injury [4]. However, neurological complications still remain a challenge in surgery employing DHCA [5]. The development of strategies to minimize cerebral injury induced by DHCA is, therefore, of tremendous clinical interest.

There is a growing body of evidence suggesting the role of inflammation and oxidative stress in neurologic dysfunction after DHCA. Kellermann et al. found that the expression of nuclear factor kappa-B (NF-κB), a vital inflammation regulator, elevates and peaks on day one after DHCA in the hippocampus of rats [6]. The production of inflammatory cytokines such as interleukin (IL)-1β, IL-6, and tumor necrosis factor-alpha (TNF-α) and reactive oxygen species (ROS) was increased in the brain tissues of DHCA model rats [7]. Zhang and colleagues demonstrated that annexin A1, a lipid mediator, protects against DHCA-induced neuron cell death in rats through the reduction in key proinflammatory cytokines by inhibiting the transcriptional activity of NF-κB [8]. A recent study in a porcine model of DHCA demonstrated that the inhibition of microglial activation, which is an index of the inflammatory response, by NO inhalation reduces neuronal degeneration [9]. The administration of hydrogen-rich saline significantly alleviates DHCA-induced brain injury through mechanisms involving antioxidative stress. The increased level of malondialdehyde and the decreased activity of superoxide dismutase (SOD) were reversed in hydrogen-rich saline-treated rats [10].

Endocannabinoids are lipid signaling molecules synthesized on demand from polyunsaturated fatty acids, which are implicated in many physiological mechanisms in the central nervous system (CNS) and peripheral tissues [11,12,13]. The endocannabinoid system consists of endocannabinoids, cannabinoid receptors, and enzymes that control the synthesis and degradation of endocannabinoids. The endocannabinoids anandamide (AEA) and 2-arachidonoylglycerol (2-AG) are synthesized in various cell types, which are able to activate typical type 1 and type 2 cannabinoid receptors (CB1R and CB2R) as well as nonclassical receptors [14].

CB1R is the brain’s most abundant G protein-coupled receptor [15], which is enriched in brain areas implicated in memory, motor coordination, and emotional processes [16,17]. Typically, the activated CB1R inhibits the activity of adenylyl cyclase (AC), the formation of cyclic adenosine monophosphate (cAMP), and the activity of protein kinase A (PKA). Several mitogen-activated protein kinases (MAPKs), including extracellular-regulated protein kinases (ERK)1/2, p38, and JNK, are activated by the CB1R, which could promote cell survival or cell death [18].

Targeting the endocannabinoid system has been shown to be neuroprotective in ischemic brain injury in both in vivo and in vitro [19,20], and the mechanisms underlying endocannabinoid-mediated neuroprotection include the attenuation of excitotoxic injury [21], the inhibition of an inflammation response [22] and oxidative stress [23], as well as the inactivation of immune cells [24]. WIN55, 212-2, a synthesized cannabinoid analog, possesses improved dissolution characteristics, unlike other cannabinoid receptor agonists. Furthermore, WIN55, 212-2 interacts negligibly with other neurotransmitter systems or ion channels [25]. The effect of WIN55, 212-2 on DHCA-induced cerebral injury remains uninvestigated.

Thus, in this study, we tested the hypothesis that WIN55, 212-2 pretreatment can alleviate cerebral injury in a rat model of DHCA, through anti-inflammation and antioxidative stress mechanisms.

## 2. Materials and Methods

### 2.1. Animals and Drug Administration

All animal experiments complied with the ARRIVE guidelines and the U.S. Public Health Service Policy on Humane Care and Use of Laboratory Animals. The study protocol was approved by the Institutional Ethics Review Board of TEDA International Cardiovascular Hospital (TICH-JY-20220715-6).

Healthy male SD rats were provided by Tianjin Auchen Laboratory Animal Company (Tianjin, China). The rats were raised in the animal center of TEDA International Cardiovascular Hospital until 8–10 months. The health conditions were in accordance with the national health standards for laboratory animals. Rearing environment: 1 week of acclimatization, adequate food and water, two rats in each cage at a temperature of 19–21 °C and 50% humidity, and 12 h of alternating light and dark. Male Sprague Dawley rats weighing between 500 and 550 g were randomly divided into three groups: a control group (*n* = 8), a DHCA group (*n* = 8), and a WIN group (*n* = 8). Rats in the control group received cardiopulmonary bypass (CPB) without DHCA. The DHCA group was subjected to CPB with DHCA. The rats in the WIN group were intraperitoneally injected with 1 mg/kg of WIN55, 212-2 (abs810695, Absin, Shanghai, China) 24 h before the CPB and DHCA procedures. The CPB and DHCA group was intraperitoneally injected with an equal volume of saline 24 h before CPB.

### 2.2. CPB and DHCA Procedures

**Figure 1:**
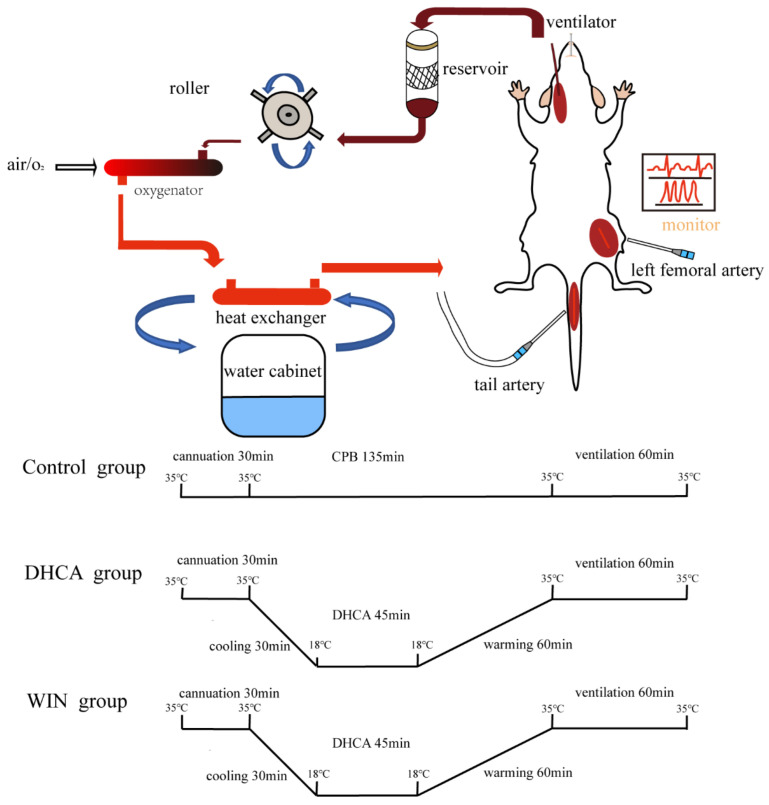
Schematic diagram of the surgical setting and experimental protocols. Control group (CPB only), DHCA group (CPB with DHCA), WIN group (WIN 55-212-2, 1 mg/kg i.p. one day before CPB with DHCA). CPB: cardiopulmonary bypass; DHCA, deep hypothermia circulatory arrest.

The CPB and DHCA procedures were performed as described in our previous study [26] with slight modifications. Briefly, after fasting for 12 h, the animals were anesthetized with 2% isoflurane. Mechanical ventilation (Alcott Biotech, Shanghai, China) with a tidal volume of 8–10 mL/kg and a respiratory rate of approximately 65 cycles per minute was started after trachea intubation. A 22G trocar was inserted into the left femoral artery for real-time monitoring of blood pressure and ECG with a small animal monitor (Harvard Apparatus, Holliston, MA, USA). A 22G trocar was inserted into the tail artery for the arterial inflow from the CPB circuit and the right internal jugular vein was cannulated for blood drainage using a homemade multiorifice catheter (Figure 1). CPB was initiated by gradually raising the flow velocity to more than 120 mL/kg/min. The nasopharyngeal temperature was cooled to 18 °C within 30 min then maintained for 45 min. After 45 min of DHCA, CPB was resumed and the nasopharyngeal temperature was increased to 34 °C within 60 min. CPB was then stopped and the mechanical ventilation was maintained for 1 h. Finally, the rats were sacrificed under deep anesthesia and the brain hemispheres were separated. Blood samples were collected after discontinuing CPB. The experimental settings are schematically illustrated in Figure 1.

### 2.3. Histopathological Study

Brain tissues fixed in 4% paraformaldehyde (abs9179, Absin, Shanghai, China) were dehydrated and embedded in paraffin according to standard histological protocols. The embedded samples were then cut into 5 μm thick transverse slices for hematoxylin–eosin (H&E) (GP1031, Servicebio, Wuhan, China) staining. Pathological scores (0 to 4) were recorded for the region of the hippocampus CA1 [10].

### 2.4. Immunofluorescence Staining of SOD

The brain slices underwent deparaffinization and antigen retrieval in sequence. The brain slices were incubated with rabbit anti-SOD antibody (1:200, Servicebio, Wuhan, China) overnight at 4 °C and then incubated with FITC-conjugated anti-rabbit IgG antibody (1:300, Servicebio, Wuhan, China) at 27 °C for 50 min. The fluorescence intensity of SOD in the hippocampus was detected using a fluorescence microscope (Nikon Eclipse C1, Nikon, Tokyo, Japan), and the results were quantified using ImageJ software. Three regions were selected randomly in the CA1 area per animal and the average intensity of immunofluorescence was measured.

### 2.5. Western Blot Analysis

Hippocampal tissues were collected for Western blotting. After homogenization, the whole-cell protein was extracted and protein concentration was determined by bicinchoninic acid assay (abs9232, Absin, Shanghai, China). The protein was electrotransferred onto a polyvinylidenedifluoride (PVDF) membrane (Millipore, USA) after being separated by 10% sodium dodecyl sulfate-polyacrylamide gel electrophoresis (SDS-PAGE, Millipore, St. Louis, MI, USA). The membrane was blocked with 5% nonfat milk for 2 h at room temperature before being incubated with one of the following primary antibodies at 4 °C overnight: caspase-3 polyclonal antibody (1:1000, 9962S), SOD polyclonal antibody (1:1000, 37385S), CB1R polyclonal antibody (1:1000, 93815S), and GAPDH were used (1:1000, 2118S). Then, the membrane was incubated with sheep anti-rabbit IgG, horseradish peroxidase-labeled secondary antibody at room temperature for 1.5 h. All of the antibodies were from Cell Signaling Technology, Danvers, MA, USA. The images were captured and analyzed using a gel analysis system (Labworks^TM^ Analysis Software, Lehi, UT, USA). Band intensities were quantified using Image J software (National Institutes of Health, Bethesda, MD, USA) and normalized with GAPDH.

### 2.6. Enzyme-Linked Immunosorbent Assay

The blood sample was centrifugated at 1000 rpm for 15 min at 4 °C and the supernatant was collected. The protein levels of TNF-α, IL-1β, IL-6, and SOD were determined with commercial ELISA kits (JL13202, JL20884, JL20896, and JL22893) from Jianglai Bioengineering Institute (Shanghai, China) according to the manufacturer’s instructions. All samples were assayed in duplicate, and the final concentrations of TNF-α, IL-1β, IL-6, and SOD were expressed as picograms per milliliter.

### 2.7. Statistical Analysis

Data are presented as mean ± standard deviations and were analyzed using one-way ANOVA using GraphPad Prism 9 software (GraphPad Software, San Diego, CA, USA), followed by Tukey’s post hoc test. *p* < 0.05 was considered statistically significant.

## 3. Results

### 3.1. WIN55, 212-2 Alleviates Hippocampal Injury in Rats Underwent DHCA

**Figure 2:**
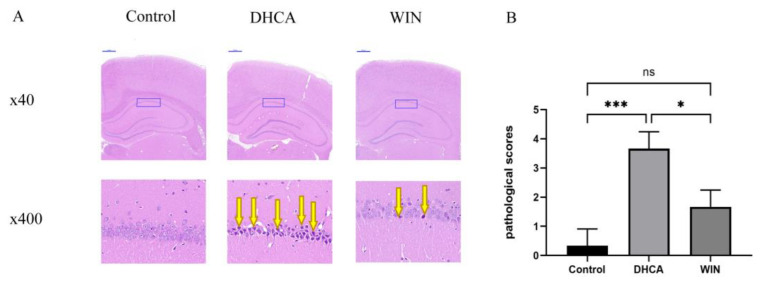
Hematoxylin–eosin staining showing the attenuation of hippocampal injury by WIN55, 212-2 in rats that underwent DHCA. (**A**) The blue boxes indicate the location of the hippocampus CA1 area. Neurons with pyknotic nuclei (yellow arrows) were decreased in the WIN55, 212-2-treated group. The scale bar is 500 μm. (**B**) Comparison of pathological scores of the hippocampus CA1. The region between the control, DHCA, and the WIN group (*n* = 3). * *p* < 0.05, and *** *p* < 0.001. ns, no significance. DHCA, deep hypothermia circulatory arrest. CPB, cardiopulmonary bypass.

H&E staining showed that in the control group, intact neurons with clear nucleoli are abundant, and arrange closely in the CA1 area of the hippocampus. In contrast, the DHCA group exhibited severely aberrant cell morphology and the nuclei of the neurons appeared to be pyknotic, which was consistent with the finding of our previous study. Treatment with WIN55, 212-2 alleviated the morphological changes in neurons in the CA1 area of the hippocampus in rats that underwent DHCA. Neurons with pyknotic nuclei were decreased in WIN55, 212-2-treated rats (Figure 2A), Rats in the DHCA group carried a significantly higher pathological score than the WIN animals (Figure 2B, *p* < 0.05).

### 3.2. WIN55, 212-2 Attenuates the Production of Proinflammatory Cytokines in Rats That Underwent DHCA

**Figure 3:**
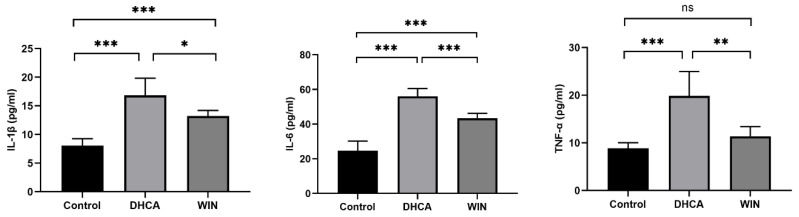
WIN55, 212-2 inhibits the elevation of circulating inflammatory cytokines in rats that underwent DHCA. *n* = 6. * *p* < 0.05, ** *p* < 0.01, and *** *p* < 0.001. ns, no significance. DHCA, deep hypothermia circulatory arrest. WIN: WIN55, 212-2.

Rats that underwent DHCA showed a significant elevation in the plasma concentration of IL-1β (16.84 ± 3.01 vs. 8.07 ± 1.20 pg/mL in control, *p* < 0.001), IL-6 (56.04 ± 4.52 vs. 24.72 ± 5.49 in control, *p* < 0.001), and TNF-α (19.87 ± 55.13 vs. 8.87 ± 1.16 in control, *p* < 0.001), which was effectively lowered by WIN55, 212-2 treatment. The plasma concentrations of IL-1β, IL-6, and TNF-α were 13.24 ± 0.95 (*p* < 0.05), 43.35 ± 2.87 (*p* < 0.001), and 11.39 ± 2.03 (*p* < 0.01), respectively, in the WIN55, 212-2-treated group (Figure 3).

### 3.3. WIN55, 212-2 Restores SOD Level in Rats That Underwent DHCA

**Figure 4:**
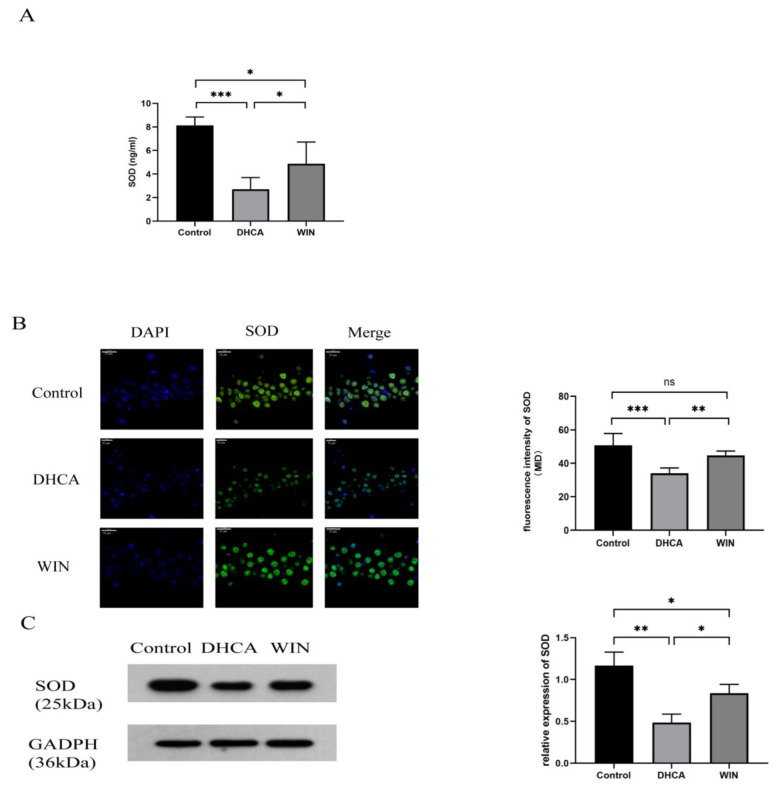
WIN55, 212-2 inhibits the decrease in SOD in rats that underwent DHCA. (**A**) Comparison of the plasma SOD levels among control, DHCA, and WIN55, 212-2-treated DHCA groups. *n* = 6. (**B**) Immunofluorescence staining showing the differences among groups in SOD content in the hippocampal tissue of the rats. Representative images: magnification 900×, bar graph: *n* = 5. The scale bar is 20 μm. (**C**) Western blot analysis of SOD expression in different groups. *n* = 3. * *p* < 0.05, ** *p* < 0.01, and *** *p* < 0.001. ns, no significance. DHCA, deep hypothermia circulatory arrest. WIN: WIN55, 212-2.

In comparison with the rats that underwent CPB only, rats subjected to CPB with DHCA showed a significant decrease in the plasma level of SOD (2.71 ± 0.10 vs. 8.14 ± 0.71 pg/mL, *p* < 0.001). Treating the rats with WIN55, 212-2 before DHCA increased the SOD content in the plasma (4.88 ± 1.84, *p* < 0.05 vs. DHCA) (Figure 4A). The loss of SOD following DHCA and the restoration by WIN55, 212-2 were also observed in the hippocampal tissue of the rats, as evidenced by the immunofluorescence staining (Figure 4B) and Western blot detection of SOD (Figure 4C).

### 3.4. WIN55, 212-2 Inhibits the Expression of Apoptotic Protein and Upregulates the Expression of CB1R in the Hippocampal Tissue of Rats That Underwent DHCA

**Figure 5:**
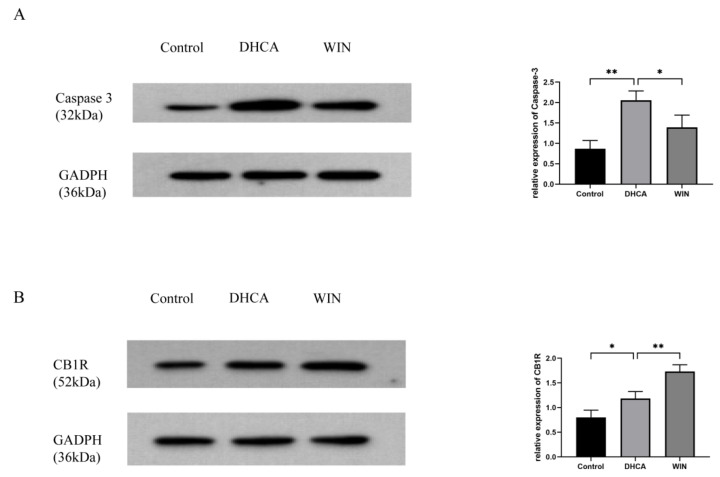
WIN55, 212-2 downregulates the expression of caspase 3 (**A**) and upregulates the expression of CB1R (**B**) in the hippocampal tissue of DHCA rats. *n* = 3. * *p* < 0.05, ** *p* < 0.01. DHCA, deep hypothermia circulatory arrest. WIN: WIN55, 212-2.

DHCA caused a significant upregulation of the apoptotic protein Caspase-3 in the hippocampus of rats, which was inhibited by the WIN55, 212-2 treatment (Figure 5A). Compared with the control group and the DHCA group, the expression of CB1R in the hippocampus of the WIN group was significantly higher, suggesting that the neuroprotection conferred by WIN55, 212-2 may be attributed to the upregulation of CB1R (Figure 5B).

## 4. Discussion

The results from the present study demonstrated, for the first time, the neuroprotective effect of cannabinoids in the DHCA model. We mainly focused on the changes in the hippocampus because it is vulnerable to hypoxic/ischemic injury. Our data showed that intraperitoneal injection of WIN55, 212-2 before DHCA significantly downregulates inflammatory cytokines and upregulates antioxidative enzymes, which are associated with a reduction in neuronal apoptosis in the rats.

The hippocampus consists of the hippocampus (also known as Ammon’s horn), the dentate gyrus, and the subiculum, playing key roles in spatial navigation and episodic memory [27,28]. Spikes fired by the hippocampal trisynaptic circuitry (dentate gyrus–CA3–CA1) have been implicated in a wide range of behaviors, including novelty recognition, pattern separation, and spatial learning [29]. The hippocampus is found vulnerable to ischemia injury and is the focus of the cerebral ischemic and reperfusion models [30].

SOD is present in the cytosol, nucleus, and mitochondrial membrane in mammalian cells, acting as an antioxidative enzyme [31]. Oxidative stress and the loss of SOD have been implicated in cerebral ischemia-reperfusion injury [32,33]. In this study, the level of SOD, both in the plasma and hippocampal tissue, was found to be decreased significantly in the DHCA group compared with the control group, which was partially reversed in the DHCA group treated with WIN55, 212-2. It has been proven that the restoration of SOD is a mechanism underlying the protective effect of cannabinoids against cerebral ischemia-reperfusion injury. For example, in a mouse model of transient middle cerebral artery occlusion, Sun et al. found that pretreatment with the CB1R agonists arachidonyl-2-chloroethylamide or WIN55, 212-2 increases the expression of MnSOD in the penumbra. Electroacupuncture pretreatment confers neuroprotection against ischemic damage through upregulating MnSOD; thus, attenuating oxidative stress via CB1R-mediated STAT3 phosphorylation. The beneficial effect of electroacupuncture pretreatment was reversed by the knockdown of MnSOD [34]. Additionally, endogenous cannabinoid anandamide-protected HT22 cells, a mouse hippocampal neuron cell line, from H_2_O_2_-induced oxidative injury. The simultaneous administration of the CB1 antagonist AM251 or CB1-siRNA abolished the preventive effect of anandamide on the increase in ROS and oxidized glutathione, and the decrease in SOD [35]. Consistent with these studies, our data showed that WIN55, 212-2 significantly increased SOD in rats that underwent DHCA. Furthermore, we found that WIN55, 212-2 increased SOD levels not only in the hippocampus but also in the plasma. Taking into account the relationship between oxidative stress and inflammation [36], the systemic elevation of the antioxidative capacity may contribute to inflammation alleviation observed in WIN55, 212-2-treated DHCA rats.

Systemic inflammation manifested by increased inflammatory cytokines in circulation after cardiac surgery is a critical cause leading to neuroinflammation [37,38]. In patients receiving aortic arch replacement with DHCA, levels of circulating inflammatory cytokines such as IL-1β, IL-6, and TNF-α were significantly elevated [39], and similar results were obtained in animal model studies of DHCA [7,40]. Results from the present study showing the increase in IL-1β, IL-6, and TNF-α in the plasma of DHCA rats are in agreement with these previous reports. Treatment with WIN55, 212-2 lowered IL-1β, IL-6, and TNF-α levels, which provided additional evidence for the anti-inflammatory property of WIN55, 212-2.

The increase in SOD and decrease in inflammatory cytokines in WIN55, 212-2-treated rats were associated with an alleviation of hippocampal destruction, as indicated by improved cell morphology and fewer neurons with pyknotic nuclei. The measurement of the apoptotic protein caspase-3 in the hippocampal tissue suggested that WIN55, 212-2 may protect neurons from DHCA-induced apoptosis. A study in fetal lambs demonstrated that WIN 55, 212-2 reduces hypoxia/ischemia-induced apoptotic cell death in the brain through the maintenance of mitochondrial integrity and functionality [41]. In our previous study, we observed ultrastructure changes in mitochondria in the hippocampus of rats following DHCA, showing swelling, cavitation of the mitochondrial matrix, and loss of the mitochondrial cristae [26]. Whether WIN 55, 212-2 preserves mitochondrial integrity and functionality to improve neuronal survival in the hippocampus is, therefore, of interest for further studies.

In the present study, we found that the expression of CB1R in the hippocampus was increased after DHCA. Previous studies of ischemic brain injury following middle cerebral artery occlusion also showed the upregulation of CB1R in the brain tissue [42,43], although the mechanisms by which ischemic insult and DHCA induce the expression of CB1R remain unclear. The increase in CB1R may facilitate the action of endogenous and exogenous cannabinoids in these pathological conditions. DHCA rats treated with WIN-55, 212-2 showed even higher expression levels of CB1R, which further highlighted the significance of the cannabinoid system in the development and treatment of ischemic brain injury.

NF-κB is an important transcription factor; the NF-κB family consists of five different DNA-binding proteins that form a variety of homodimers and heterodimers [44]. The activity of NF-κB is regulated by the level of intracellular oxidative stress, as well as the level of inflammation [45]. The NF-κB signal is activated in some pathological conditions. For instance, activated NF-κB translocates to the nucleus, stimulating the secretion of inflammatory factors such as IL-1β, TNF-α, and IL-6, thus aggravating the inflammatory response after infection [46,47]. In an animal model of spinal cord ischemic injury, the cytoplasmic protein expressions of NF-κB were decreased when treated with WIN55, 212-2 [48]. In addition, the NF-κB signal pathway was proven to be a significant target to mitigate brain injury in a rat model of DHCA [49]. Therefore, we deduce that the NF-κB signal pathway might be regulated by the endocannabinoid system in the DHCA condition. This hypothesis needs to be proven in future studies.

From the current studies, most FDA-approved cannabinoids (e.g., Epidiolex, Cesamet, and Marinol) in humans are used as antiepileptics, antiemetics, or analgesics in cancer patients. Although the impact of cannabinoids in experimental stroke has been wildly proven both in vivo and in vitro, the clinical trials for cannabinoids are merely limited to neurodegenerative diseases [50,51]. The possible reasons are as follows: (1) because cannabinoid receptors are widely distributed in the brain and have a proven involvement with the addictive system [52]; (2) cannabinoid receptors are not only present in the central nervous system but also widely distributed throughout the body and are associated with cardiovascular diseases, skin disorders, and tumor diseases [53,54,55]; therefore, a comprehensive evaluation is needed before clinical trials; and (3) the involvement of the endocannabinoid system in the physiological functions and the pathological state of diseases has allowed the development of more efficacious and safer cannabinoid-based drugs [56]. Further research is needed to identify promising therapeutic targets within the endocannabinoid system and to investigate the pharmacological effects of cannabinoids.

Several limitations of this study should be noted. Firstly, although we provided evidence showing the protective effect of WIN-55, 212-2 on the hippocampus in rats after DHCA, no functional assessment of the hippocampus was conducted, which requires further studies on the long-term cognitive outcome of DHCA. In addition, the impact of WIN55, 212-2 treatment on other organs in DHCA rats needs to be clarified. Secondly, we only examined the expression of CB1R due to its predominance in the brain; further evaluation of CB2R shall help complete the picture of the impact of DHCA on CB receptors. Thirdly, in vitro studies employing cell models of DHCA are warranted to unravel the signal transduction involved in the antiapoptotic action of WIN55, 212-2 in the brain.

## 5. Conclusions

In summary, the administration of WIN55, 212-2 alleviates hippocampal injury induced by DHCA in rats by regulating intrinsic inflammatory and oxidative stress responses through a CB1R-dependent mechanism. These results support the potential use of WIN55, 212-2 in DHCA-related brain injury.

## Data Availability

Data are available on request due to privacy restrictions.

## Abbreviations

DHCA	deep hypothermic circulatory arrest
CB1R	type 1 cannabinoid receptor
CB2R	type 2 cannabinoid receptor
CPB	cardiopulmonary bypass
IL	interleukin
TNF-α	tumor necrosis factor-alpha
SOD	superoxide dismutase
ROS	reactive oxygen species
GAPDH	glyceraldehyde-3-phosphate dehydrogenase
ELISA	enzyme-linked immunosorbent assay
MID	mean immunofluorescence density
CNS	central nervous system
AEA	anandamide
2-AG	2-arachidonoylglycerol
NF-κB	nuclear factor kappa-B
AC	adenylyl cyclase
MAPKs	mitogen-activated protein kinases
PKA	protein kinase A
ERK	Extracellular-regulated protein kinases
JNK	Jun N-terminal kinase
